# Anticonvulsant Properties of 1-Diethylamino-3-phenylprop-2-en-1-one

**DOI:** 10.3390/medicines10090054

**Published:** 2023-09-08

**Authors:** Swagatika Das, Praveen K. Roayapalley, Sarvesh C. Vashishtha, Umashankar Das, Jonathan R. Dimmock

**Affiliations:** Drug Discovery and Development Research Group, University of Saskatchewan, Saskatoon, SK S7N 5E5, Canadajr.dimmock@usask.ca (J.R.D.)

**Keywords:** anticonvulsant, antiepileptic, seizures, anticonvulsant screens, neurotoxicity, convulsant

## Abstract

There is a need for novel antiepileptic agents whose modes of action differ from those of current antiepileptic drugs. The objective of this study was to determine whether 1-diethylamino-3-phenylprop-2-en-1-one (**2**) could prevent or at least diminish convulsions caused by different mechanisms. This amide afforded protection in the maximal electroshock and subcutaneous pentylenetetrazole screens when given intraperitoneally to both mice and rats. A number of specialized tests in mice were conducted and are explained in the text. They revealed (**2**) to have efficacy in the 6 Hz psychomotor seizure test, the corneal kindling model, the mouse temporal epilepsy screen and a peripheral neuronal transmission test using formalin. Three screens in rats were undertaken, which revealed that (**2**) blocked chloride channels, inhibited peripheral neuronal transmission (tested using sciatic ligation and von Frey fibres) and afforded protection in the lamotrigine-resistant kindled rat model. The biodata generated reveal that (**2**) is an important lead molecule in the quest for novel structures to combat epilepsy.

## 1. Introduction

A large number of people who suffer from epilepsy do not have drugs that deal with this problem adequately [1,2]. Hence, the quest to develop novel compounds to treat epilepsy is crucial. Various antiepileptic drugs contain an aryl ring and an amidic group, which are in a fixed relationship with each other. A number of antiepileptic drugs contain one or more aryl rings and amidic groups, as illustrated in the structure of phenytoin [3,4] and carbamazepine [3]. The freedom of motion of different groups is reduced in these compounds as well as (**1**) and (**2**). In light of this observation, several years ago, the decision was made in our laboratories to prepare series **1** as candidate antiepileptic agents, in which the aryl ring and amidic group are held in a specific location by both the rigid olefinic group and the piperidine ring [5].

**Figure 1:**
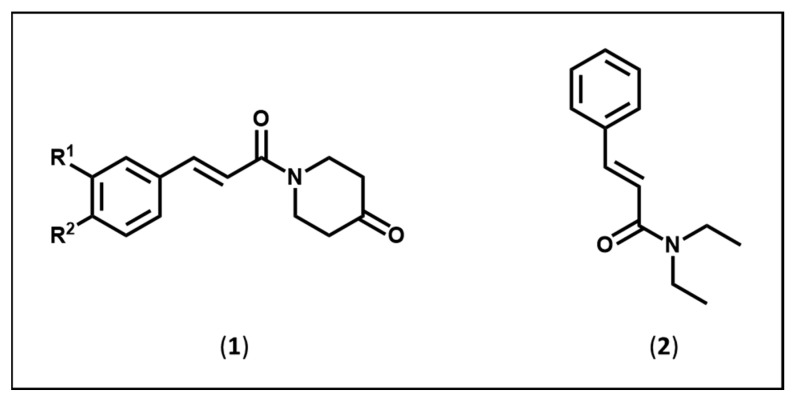
Structures of the amides in series (**1**) and (**2**).

The evaluation of candidate anticonvulsants often commences with the intraperitoneal administration of varying doses of the compound to mice and undertaking three bioevaluations as follows. The maximal electroshock (MES) screen is alleged to predict compounds with efficacy against generalized tonic–clonic seizures, while the subcutaneous pentylenetetrazole (scPTZ) test is thought to detect compounds useful in treating myoclonic seizures [6]. In addition, neurotoxicity (TOX) may be evaluated using the rotarod test [7]. In general, the compounds in series (**1**), and especially (**1a**) (R^1^ = R^2^ = H), display anticonvulsant properties in both the MES and scPTZ screens as well as displaying some neurotoxicity [5]. In view of this observation, the molecular simplification of (**1a**) was undertaken, leading to (**2**) in order to assess whether the piperidine ring with a keto group was a structural ballast (as indicated in Figure 1) or not. After the intraperitoneal injection of (**2**) in mice, the ED_50_ values in the MES and scPTZ screens and the TD_50_ evaluation were 52.0, 69.4 and 114 mg/kg, respectively [5]. The anticonvulsant data compare favourably with valproic acid, for example, with ED_50_ values for the latter in the MES and scPTZ screens of 263 and 220 mg/kg, respectively, and a TD_50_ value of 398 mg/kg [6]. Thus, (**2**) is a lead molecule, and further screening was undertaken.

After oral administration to rats, (**2**) had an ED_50_ value in the MES screen of 25.3 mg/kg. In addition, 2/2 rats who received a dose of 225 mg/kg of (**2**) were protected in the scPTZ screen. No toxicity was noted using doses up to and including 450 mg/kg. Furthermore, (**2**) was evaluated in the hippocampal kindling rat model using a literature procedure [8]. This screen is used to detect compounds that are effective in treating complex partial seizures. The dose of (**2**) administered via the intraperitoneal route, which is required to protect half of the rats against seizures, in this model was 43.1 mg/kg and, in addition, the time of after-discharge duration was reduced [5].

These results with (**2**) mentioned above were disclosed a number of years ago [5]. The purpose of the current report is to outline further experimentation, which has been undertaken with (**2**) in order to evaluate its potential as a candidate antiepileptic drug.

## 2. Materials and Methods

### 2.1. Synthesis of Compound (***2***)

Compound (**2**) was synthesized following a literature procedure [5].

### 2.2. Bioassays

The bioevaluations of (**2**) were conducted in the Epilepsy Therapy Screening Program (ETSP) of the National Institute of Neurological Disorders and Stroke, USA. The methodology followed in conducting these preliminary screens is available on the internet [9], while details may also be found in different published works [10,11,12]. The ETSP has been in place for over 40 years and provides a screening service for researchers in the USA and abroad. It has over 30,000 compounds in its internal program database and has contributed to the development of several FDA-approved drugs. Detailed descriptions of the testing methods used for screening are provided in its PANACHE (Public Access to Neuroactive and Anticonvulsant Chemical Evaluations) website. A number of compounds prepared in our laboratory have been assessed for antiepileptic properties using the ETSP protocol [5,13,14]. The purpose of the bioassays is to determine whether compound (**2**), identified in an earlier investigation as a possible antiepileptic agent, would demonstrate antiepileptic properties towards different types of epilepsy.

## 3. Results

As shown in Table 1, compound (**2**) afforded protection in the MES and scPTZ screens and had a TD_50_ of 82.4 mg/kg after intraperitoneal administration in rats. This molecule also afforded protection in the 6 MHz psychomotor seizure test after both intraperitoneal and oral administration in mice. Compound (**2**) also afforded protection in the corneal kindling model (CKM) in mice. Further protection was afforded in the mouse temporal lobe epilepsy screen, while in the formalin test, (**2**) reduced peripheral neural transmission. This compound interacted with chloride channels (pilocarpine test) and blocked peripheral neuronal transmission in rats (measured using von Frey fibres). In addition, (**2**) displayed activity in the lamotrigine-resistant kindled rat model.

## 4. Discussion

The first determination was designed to evaluate the efficacy of (**2**) in the MES, scPTZ and TOX screens after intraperitoneal injection in rats (Table 1). This experimentation was undertaken for two reasons: first, to determine if there is a difference in the efficacy of (**2**) in rats and mice, i.e., whether a species-specific response was observed; second, to compare the anticonvulsant properties of (**2**) in rats after administration via both the intraperitoneal and oral routes. The ED_50_ and TD_50_ values of (**2**) in the MES, scPTZ and TOX screens after intraperitoneal administration to mice were 52.0, 69.4 and 114 mg/kg, respectively [5], while the biodata using rats are presented in Table 1. The anticonvulsant potency of (**2**) is 2.52- and 2.05-times greater in the MES and scPTZ screens, respectively, when administered to rats compared to mice, while neurotoxicity is not significantly different. The previous investigation revealed that in the MES and TOX screens, the ED_50_ and TD_50_ values of (**2**) were 25.3 and >450 mg/kg, respectively, when administered orally to rats [5]. Hence, the potency of (**2**) in the MES screen in rats is the same whether administered intraperitoneally or orally to rats. On the other hand, toxicity is greater when (**2**) is given via the intraperitoneal route.

These results indicated that additional evaluation of (**2**) in a number of advanced screens was warranted, and those screens are described below.

The 6 Hz psychomotor seizure test was introduced to detect compounds that may be useful in treating drug-resistant partial seizures [15]. This screen differs from the MES bioassay insofar as it uses a lower frequency (6 Hz vs. 60 Hz), less current (32 or 44 mA vs. 150 mA) and a longer challenge (3 s vs. 0.2 s). The intraperitoneal administration of (**2**) to mice revealed that in the 6 Hz screen using a current of 44 mA, the ED_50_ value was 95.8 mg/kg (Table 1). This result may be compared with the ED_50_ values of valproic acid and phenobarbital of 126 (94.5–152) and 14.8 (8.9–23.9) mg/kg, respectively [13]. One may conclude that in this test, (**2**) is equipotent with valproic acid but is less effective than phenobarbital.

The decision was made to determine whether efficacy in the 6 Hz screen would be encountered if (**2**) was administered orally to mice. This experiment was initiated for two reasons. First, antiepileptic drugs are generally administered over long periods of time and, hence, the preferred route of administration is oral. Second, the result should indicate whether the route of administration affects anticonvulsant potency. Using a current of 44 mA, the ED_50_ value was 145 mg/kg (Table 1), which is statistically indistinguishable from the ED_50_ value of 95.8 mg/kg when (**2**) is administered intraperitoneally. Thus, the evidence to hand reveals that (**2**) may be useful in treating a number of different types of epilepsy.

The next screen used was the corneal kindling model (CKM) in mice. Kindling is a process whereby repeated seizures lead to an increase in the number of seizures that occur. The CKM screen identifies compounds, which block fully kindled seizures and, thus, may be of use clinically in treating human partial epilepsy. The intraperitoneal injection of (**2**) in mice revealed that after 0.25 h, the ED_50_ value of this compound in this screen was 50.1 mg/kg (Table 1), which, taking into consideration its activity in the hippocampal kindling rat model (ED_50_ of 43.1 mg/kg), strongly suggests that it would have utility in treating human partial epilepsy.

A screen was devised to identify compounds that are effective against temporal lobe epilepsy (TLE) in humans. It is carried out in mice, and this test is known as the mouse temporal lobe epilepsy (MTLE) screen. In this model, kainic acid was injected into the dorsal hippocampus of mice and, four weeks later, (**2**) was injected intraperitoneally into the animals. The hippocampus paroxysmal discharge (HPD) figures were obtained in treated and untreated mice. Over a period of 20 min, the number of HPDs in four untreated mice was 11, 21, 13 and 13. During the same time frame, after the administration of 80 mg/kg of (**2**), the number of HPDs in four mice was 2, 5, 3 and 24. Thus, (**2**) was effective in three of four animals in the MTLE screen.

The pilocarpine screen was carried out using rats. Pilocarpine is a convulsant, which blocks the GABA_A_ receptor-activated chloride ionophore. Hence, the compounds which counteract the convulsant properties of pilocarpine exert their properties, in part at least, through interaction with chloride channels. Compound (**2**) was administered intraperitoneally to rats immediately after the first stage III seizure. Using a dose of 65 mg/kg of (**2**), 7/7 animals were protected.

In another experiment, (**2**) was assessed for its activity in the lamotrigine-resistant kindled rat model. This assay was designed to find compounds with novel mechanisms of action and potential for treating medically resistant seizures. When administered intraperitoneally, after 0.25 h, (**2**) had an ED_50_ value of 62.5 mg/kg (Table 1). Using an intraperitoneal dose of 70 mg/kg, the seizure score of 5 in the control animals was reduced to 1.88 ± 0.77 (mean ± SEM) and the after-discharge duration from 112.75 ± 13.16 s to 56.5 ± 22.46 s (means ± SD).

Since pain is often comorbid with epilepsy [16] and several anticonvulsants are used in treating pain [17,18], two screens for pain were also included, one in mice and one in rats. Administration of a compound with both antiepileptic and analgesic properties could eliminate the need for using several drugs with varying pharmacokinetic properties. A formalin test in mice evaluates the ability of a compound to inhibit peripheral neuronal transmission. In this screen, the amount of time the animals spend licking an afflicted hind paw within a 2-min period is recorded at 5-min intervals for 40 min. This method produces a biphasic response, and the area under the curve reveals the acute and inflammatory stages. In the case of (**2**), a dose of 52 mg/kg was administered intraperitoneally into mice; the dose chosen was based on the ED_50_ value of (**2**) in the MES screen being 52 mg/kg vide supra. In the acute phase, the reduction in licking was 48% (*p* < 0.05 compared to controls) but 56% in the inflammatory stage (*p* < 0.05 compared to controls).

The amide (**2**) was also examined for its capacity to inhibit peripheral neuronal transmission using the sciatic ligation model in rats. In this experiment, calibrated von Frey fibres are placed in the hind paws of the animals. If a positive response is noted, i.e., withdrawal of the foot, a weaker fibre is applied, and the process is repeated until the 50% threshold for withdrawal in grams is noted. Then, a dose of 40 mg/kg of (**2**) was administered intraperitoneally, and the time to peak effect was determined and found to be **2** hours. At this point in time, a statistically significant increase in the threshold was noted, namely 180% compared to the control (100%). Thus, (**2**) blocks the neuropathic pain caused by sciatic nerve ligation.

The evidence presented in this study (coupled to the results generated in a preliminary investigation [5]) reveals (**2**) to be a promising lead compound. This amide afforded protection in the MES and scPTZ screens when administered intraperitoneally to both rats and mice. A number of specialized tests were conducted with (**2**) to explore its potential usefulness in treating different types of epileptic seizures, namely drug-resistant partial seizures (6 Hz test), human partial epilepsy (corneal kindling model) and temporal lobe epilepsy, as well as pain (peripheral neuronal transmission-formalin test). In these screens, (**2**) was administered intraperitoneally to mice. Several additional tests were conducted in rats when (**2**) was given via the intraperitoneal route. The amide (**2**) is a chloride channel blocker (pilocarpine test), has efficacy in inhibiting neuronal transmission (measured using von Frey’s fibres) and should prove useful in treating medically resistant seizures (lamotrigine-resistant kindled rat model). At the same time, future bioevaluations of (**2**) should be conducted, including additional mode-of-action studies, effects of (**2**) on drug-metabolizing enzymes and its efficacy using other animal models as well as undertaking toxicity studies.

In summary, this study revealed that (**2**) is an excellent lead molecule for developing novel anticonvulsants. Recently, the activity of (**2**) in the initial MES screen was confirmed [19], and analogues of (**2**) have demonstrated anticonvulsant properties [20,21].

## 5. Conclusions

This study provided ample evidence that (**2**) is an outstanding lead molecule in the campaign to combat epilepsy. In the future, molecular modifications of (**2**) should be undertaken with a view to creating structure–activity and quantitative structure–activity relationships by placing various substituents in the aryl ring followed by undertaking the appropriate bioassays. Reduction of both the olefinic double bond and carbonyl group in (**2**) will lead to an analogue now, which lacks the capacity to interact with cellular nucleophiles. Replacement of the piperidino ring with other acyclic and aliphatic amines may lead to compounds with analogues with significantly greater anticonvulsant potencies.

## Figures and Tables

**Table 1:** Quantitative anticonvulsant data for (**2**).

Animals	Route of Administration ^a^	Time (h)	Screen ^b^	ED_50_ or TD_50_ (mg/kg) (95% CI)
Rats	IP	0.25	MES	20.7 (14.15–25.05)
Rats	IP	0.25	scPTZ	33.9 (25.88–46.28)
Rats	IP	0.25	TOX	82.4 (64.08–102.82)
Mice	IP	0.25	6 Hz	95.8 (66.67–131.12)
Mice	PO	0.50	6 Hz	144.6 (90.89–205.50)
Mice	IP	0.25	CKM	50.1 (31.50–68.80)
Rats	IP	0.25	LTG-R	62.5 (56.62–70.40)

^a^ The letters IP and PO refer to the administration of (**2**) by the intraperitoneal and oral routes, respectively. ^b^ The abbreviations refer to the maximal electroshock (MES), subcutaneous pentylenetetrazole (scPTZ), neurotoxicity (TOX), 6 Hertz (6 Hz), corneal kindling model (CKM) and lamotrigine-resistant (LTG-R) model.

## Data Availability

The data presented in this study are available in article.

## References

[1] Riva A., Golda A., Balagura G., Amadori E., Vari M.S., Piccolo G., Iacomino M., Lat-tanzi S., Salpietro V., Minetti C. (2021). New Trends and Most Promising Therapeutic Strategies for Epilepsy Treatment. Front. Neurol..

[2] Löscher W., Potschka H., Sisodiya S.M., Vezzani A. (2020). Drug Resistance in Epilepsy: Clinical Impact, Potential Mechanisms, and New Innovative Treatment Options. Pharmacol. Rev..

[3] Grover G., Pal R., Bhatia R., Yar M.S., Nath R., Singh S., Raj K., Kumar B., Akhtar M.J. (2022). Design, synthesis, and pharmacological evaluation of aryl oxadiazole linked 1,2,4-triazine derivatives as anticonvulsant agents. Med. Chem. Res..

[4] Sahu M., Siddiqui N., Naim M., Alam O., Shahar Yar M., Sharma V., Wakode S. (2017). Design, Synthesis, and Docking Study of Pyrimidine-Triazine Hybrids for GABA Estimation in Animal Epilepsy Models: Pyrimidine-Triazine Hybrids as Anticonvulsants. Arch. Pharm..

[5] Dimmock J.R., Gunda S.G.R., Vashishtha S.C., Zello G.A., Das U., Nienaber K.H., Stables J.P., Allen T.M., Santos C.L. (2004). Anticonvulsants containing the *N*-(3-aryl-2-propenyl)amido pharmacophore. J. Enz. Inhib. Med. Chem..

[6] White H.S., Woodhead J.H., Wilcox K.S., Stables J.P., Kupferberg H.J., Wolf H.H., Levy R.H., Mattson R.H., Meldrum B.S., Perucca E. (2002). Discovery and Preclinical Development of Antiepileptic Drugs. Antiepileptic Drugs.

[7] Dunham M.S., Miya T.A. (1957). A note on a simple apparatus for detecting neurological deficit in rats and mice. J. Am. Pharm. Sci. Ed..

[8] Lothman E.W., Williamson J.M. (1994). Closely spaced recurrent hippocampal seizures elicit two types of heightened epileptogenesis: A rapidly developing, transient kindling and a slowly developing, enduring kindling. Brain Res..

[9] National Institute of Neurological Disorders and Stroke (NINDS) (2022). Public Access to Neuroactive & Anticonvulsant Chemical Evaluations (PANAChE). https://panache.ninds.nih.gov/Home/CurrentModels.

[10] Pernici C.D., Mensah J.A., Dahle E.J., Johnson K.J., Handy L., Buxton L., Smith M.D., West P.J., Metcalf C.S., Wilcox K.S. (2021). Development of an antiseizure drug screening platform for Dravet syndrome at the NINDS contract site for the Epilepsy Therapy Screening Program. Epilepsia.

[11] Wilcox K.S., West P.J., Metcalf C.S. (2020). The current approach of the Epilepsy Therapy Screening Program contract site for identifying improved therapies for the treatment of pharmacoresistant seizures in epilepsy. Neuropharmacology.

[12] Kehne J.H., Klein B.D., Raeissi S., Sharma S. (2017). The National Institute of Neurological Disorders and Stroke (NINDS) Epilepsy Therapy Screening Program (ETSP). Neurochem. Res..

[13] Vashishtha S.C., Zello G.A., Nienaber K.H., Balzarini J., De Clercq E., Stables J.P., Dimmock J.R. (2004). Cytotoxic and anticonvulsant aryloxyaryl Mannich bases and related compounds. Eur. J. Med. Chem..

[14] Dimmock J.R., Vashishtha S.C., Stables J.P. (2000). Ureylene anticonvulsants and related compounds. Pharmazie.

[15] Toman J.E.P., Everett G.M., Richards R.M. (1952). The search for new drugs against epilepsy. Tex. Rep. Biol. Med..

[16] Ottman R., Lipton R.B., Ettinger A.B., Cramer J.A., Reed M.L., Morrison A., Wan G.J. (2011). Comorbidities of epilepsy: Results from the Epilepsy Comorbidities and Health (EPIC) survey. Epilepsia.

[17] Gilron I., Baron R., Jensen T. (2015). Neuropathic pain: Principles of diagnosis and treatment. Mayo Clin. Proc..

[18] Tomic M., Pecikoza U., Micov A., Vuckovic S., Stepanaovic-Petrovic R. (2018). Antiepileptic drugs as analgesics/adjuvants in inflammatory pain: Current preclinical evidence. Pharmacol. Therap..

[19] Guan L.-P., Sui X., Deng X.-Q., Zhou D.-H., Qu Y.-L., Quan Z.S. (2011). *N*-Palmitoylethanolamide derivatives: Synthesis and studies on anticonvulsant and antidepressant activities. Med. Chem. Res..

[20] Gunia-Krzyzak A., Zeslowska E., Stoczynska K., Koczurkiewicz P., Nitek W., Zelaszczyk D., Szkaradek N., Waszkielewicz A.M., Pekala E., Marona H. (2016). Anticonvulsant activity, crystal structures and preliminary safety evaluation of N-trans-cinnamoyl derivatives of selected (un)modified aminoalkanols. Eur. J. Med. Chem..

[21] Gunia-Krzyzak A., Bareyre F.M., Marona H., Waskielewicz A.M. (2019). Four *N*-(E-cinnamoyl (cinnamide) derivatives of aminoalkanols with promising anticonvulsant and analgesic activity. Bioorg. Med. Chem. Lett..

